# Amplitude-Integrated Electroencephalography: A Readily Available Tool for Neonatologists

**DOI:** 10.7759/cureus.67018

**Published:** 2024-08-16

**Authors:** Michelle H Lucena, Palanikumar Balasundaram, Shu-wei Hsu, Diosely C Silveira, Orna Rosen

**Affiliations:** 1 Department of Pediatrics, Division of Neonatology, Baylor College of Medicine, Houston, USA; 2 Department of Pediatrics, Division of Neonatology, Mercy Health - Javon Bea Hospital, Rockford, USA; 3 Department of Neurology, Mount Sinai Health System, Icahn School of Medicine at Mount Sinai, New York City, USA; 4 Department of Neurology, University of Texas Medical Branch, Galveston, USA; 5 Department of Pediatrics, Division of Neonatology, Children’s Hospital at Montefiore, Albert Einstein College of Medicine, Bronx, USA

**Keywords:** neonate, therapeutic hypothermia, amplitude-integrated electroencephalography (aeeg), seizures, hypoxic-ischemic encephalopathy (hie)

## Abstract

Hypoxic-ischemic encephalopathy (HIE) is a common condition occurring at birth, impairing central nervous system function. Therapeutic hypothermia is beneficial for suspected HIE as it reduces mortality and disability in survivors but not for other types of encephalopathy (e.g., metabolic). Amplitude-integrated electroencephalography (aEEG) complements limited resource Neonatal Intensive Care Units as a screening tool that can provide information regarding the degree of encephalopathy and electrographic seizures. Patients with HIE are at increased risk for seizures, which are subclinical in half of the cases. The aEEG emphasizes electroencephalographic amplitude differences, whereas continuous video electroencephalography (cEEG) provides a high-resolution picture of cerebral electrical activity, making it the most accurate method for detecting subclinical seizures. Still, its interpretation demands extensive training beyond the scope of neonatologists. Any infant in whom aEEG is suspicious for seizures should undergo cEEG to confirm the findings because even very low-amplitude artifacts might be misdiagnosed as seizures. We report a case and review the utility of aEEG in detecting subclinical seizures in neonates with HIE during therapeutic hypothermia while cEEG is not available.

## Introduction

Hypoxic-ischemic encephalopathy (HIE) is a common condition caused by several disorders that impair central nervous system function, leading to transient or permanent cerebral dysfunction [1]. Therapeutic hypothermia (TH) is beneficial for suspected HIE as it reduces mortality and disability in survivors but not for other types of encephalopathy (e.g., metabolic). The benefits of survival and neurodevelopment outcomes of TH outweigh its short-term adverse effects. It is the only proven clinical therapy to ameliorate brain injury for neonates with moderate to severe HIE [2,3]. Patients with HIE are at increased risk for acute provoked seizures, and about half of their seizures during TH are subclinical and cannot be detected without neuromonitoring [4]. Amplitude-integrated electroencephalography (aEEG) uses simplified cerebral function monitoring with fewer electrodes and generates a compressed tracing, different from cEEG, which has a full complement of scalp electrodes [5]. Amplitude-integrated EEG provides information regarding functional brain abnormalities, degree of encephalopathy, and electrographic seizures [6]. It can also predict adverse outcomes when it remains severely abnormal 48 hours into therapy [7]. Here, we present a case of a neonate in whom an aEEG aided in detecting subclinical seizures, demonstrating the utility of aEEG in neonatal practice, particularly during TH.

This article was previously presented as a meeting abstract and poster at the American Epilepsy Society (AES) meeting in December 2022 in Nashville, TN. 

## Case presentation

A 41-week-old female neonate was born via emergent cesarean section due to a non-reassuring fetal heart rate to a healthy 34-year-old mother with adequate prenatal care and positive Group B Streptococcus (GBS) screening. The neonate emerged non-vigorous, hypotonic, and without any respiratory effort. The infant received positive pressure ventilation and was intubated within the first minutes of life. Apgar scores were 3, 4, and 7 at one, five, and 10 minutes of life, respectively.

This neonate is the second child of non-consanguineous parents and has one sibling with autism spectrum disorder. There is no family history of genetic disorders or seizures. The infant's facial features were non-dysmorphic, and according to the World Health Organization growth curves, the infant was large for gestational age with head circumference in the 99th percentile [8]. Initial neurologic examination was significant for mild hypotonia and hypopnea, which transitioned to a hyper-alert state with jitteriness, irritability, and agitation compatible with a Sarnat stage 1 [9]. Soon after admission, the infant presented with intermittent stiffening of both arms and legs, and a loading dose of Phenobarbital was administered. No cord gases were available, but initial arterial blood gas showed a pH of 7.15, bicarbonate of 9.1 mmoL/L, and a base deficit of 18.2 mmoL/L.

**Figure 1:**
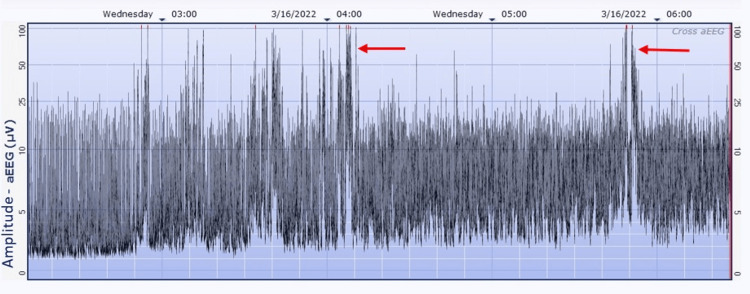
aEEG with a discontinuous pattern, moderately abnormal, suggestive of intermittent electrographic seizures (red arrows) Impedance tracing not included in the image. aEEG - Amplitude-integrated electroencephalography

Given neonatal encephalopathy with concerns for hypoxic-ischemic etiology, fetal distress, and GBS-positive status, a partial sepsis workup was performed, including a complete blood count and blood culture, and she was started on antibiotics. The neonate was transferred to the Level 4 NICU for TH within the six-hour window, and a two-channel amplitude-integrated EEG (Natus, Seattle, WA - CFM Olympic Brainz Monitor, v3.1.5) was placed as continuous video EEG was not readily available. Within the first few hours of treatment, the aEEG pictured a discontinuous pattern, moderately abnormal, suggestive of intermittent electrographic seizures (Figure 1). Clinically, there were no jerking movements or stiffening of the limbs, and her level of consciousness alternated between sleepiness and periods of agitation. The findings on the aEEG led to a second dose of Phenobarbital. 

On the second day of life, she was extubated to nasal continuous positive airway pressure as respiratory effort improved. Coagulation studies were mildly abnormal, with an international normalized ratio of 1.3 and a D-dimer of 8.58 mg/mL, but liver function was normal. Initial head ultrasonography showed a slightly increased echotexture of the thalami and lentiform nuclei. All antimicrobials were discontinued after 72 hours since the bacterial culture was negative.

**Figure 2:**
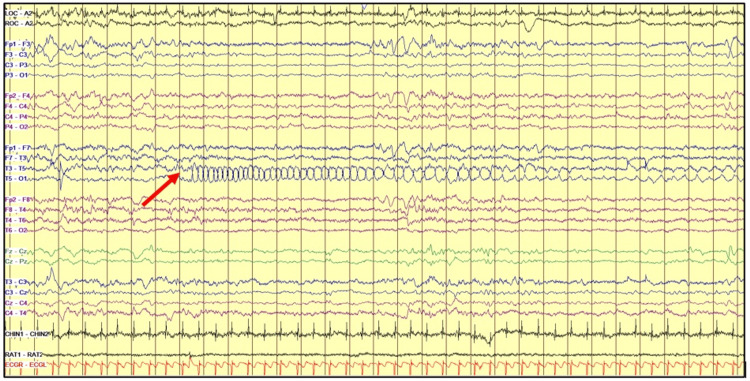
cEEG with left posterior-temporal seizure cEEG - continuous video electroencephalography

**Figure 3:**
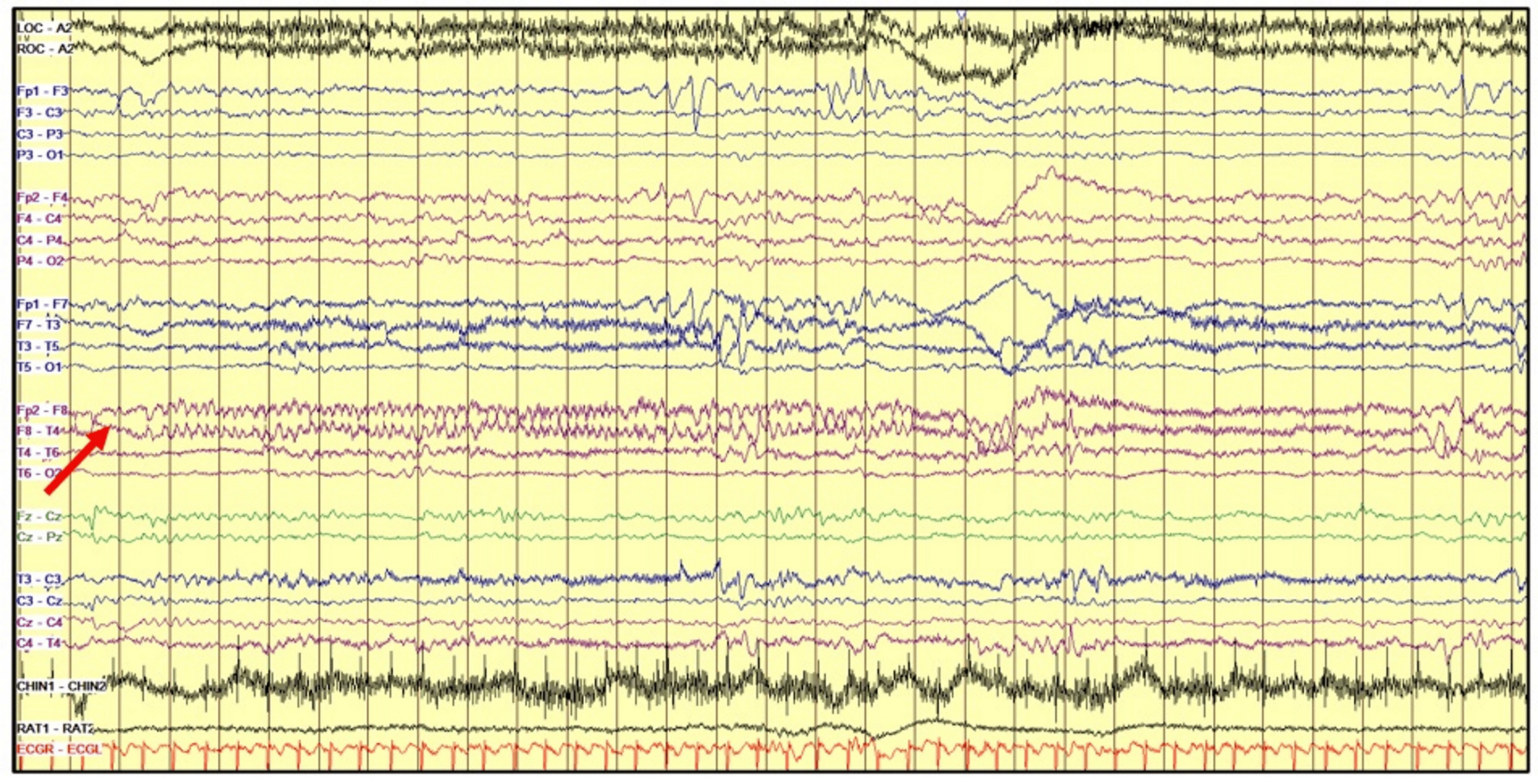
cEEG with right frontotemporal seizure cEEG - continuous video electroencephalography

Soon after admission, the Neurology team was consulted, and cEEG was started, using the international 10-20 system modified for newborns, the following day after the aEEG intra-scalp leads were removed. The cEEG showed two electrographic seizures, without evident clinical signs, arising from the left posterior-temporal and right frontotemporal regions, respectively, lasting less than 30 seconds each (Figures 2, 3).

On the sixth day of life, brain magnetic resonance imaging (MRI) showed bilateral thin subdural hematomas lying along the tentorium and bilateral posterior parietal regions. Single-voxel magnetic resonance spectroscopy over the territory of the left basal ganglia and left corona radiata demonstrated increased choline-to-creatinine metabolite ratios with decreased N-acetyl aspartate metabolite.

The patient presented a clinical seizure with horizontal nystagmus, flexion of arms, and desaturations on the eighth day of life, and Levetiracetam was added. Subsequent cEEGs were abnormal, with excessive sharp transients over the left more than right temporal regions, but no other clinical or electrographic seizures were recorded.

**Figure 4:**
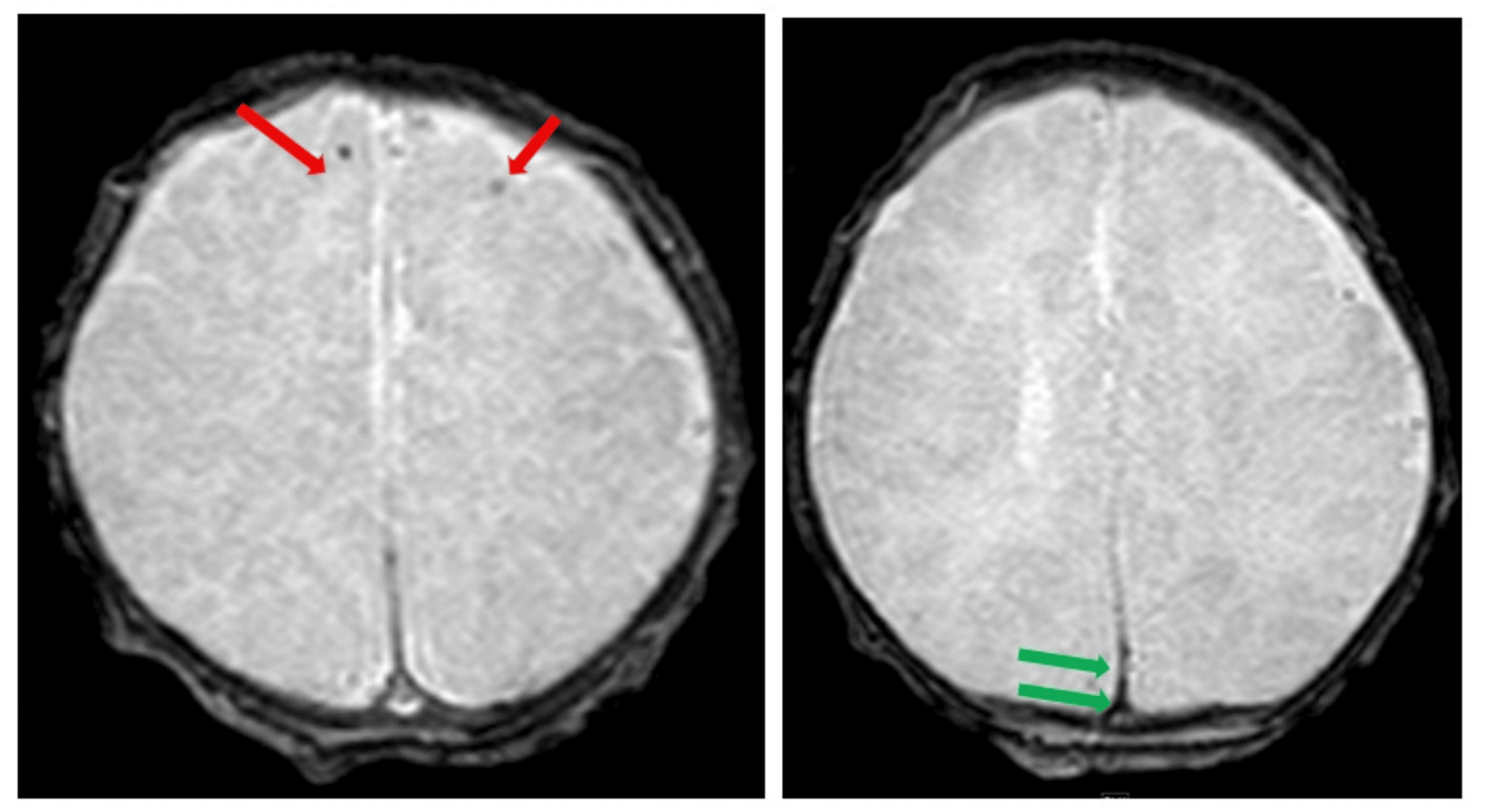
Brain MRI shows few punctate foci of susceptibility-related signal loss along the surface of the bilateral frontal lobes (left; red arrows), along the posterior falx cerebra (right; green arrows), and bilateral tentorial leaflets

A repeated brain MRI 15 days later was remarkable for a few punctate foci of susceptibility-related signal loss along the surface of the bilateral frontal lobes, along the posterior falx cerebra, and bilateral tentorial leaflets, which may represent areas of prior hemorrhage (Figure 4). Around the same time, Levetiracetam was weaned off and discontinued without recurrence of clinical or electrographic seizures.

The abnormal cEEG, the finding of previous hemorrhages on MRI, and the increased risk of seizure prompted the continuation of Phenobarbital. At three weeks of life, oral feeding successfully started. However, as she was still presenting disengagement and fatigue during feeds, she was transferred to a rehabilitation facility in room air, mainly for feeding therapy.

## Discussion

Continuous EEG monitoring is the most reliable and accurate method for detecting subclinical seizures. However, interpreting cEEG requires years of training and practice, which is outside the scope of the neonatologist. Many neonatal intensive care units (NICUs) are not equipped with a cEEG team, including technicians and neurologists, or cEEG would only be started the following day after admission. In contrast, aEEG is a readily available monitoring and diagnostic tool in many NICUs, and it has variable sensitivity among studies in the literature [10]. However, any infant in whom aEEG is suspicious for seizures should undergo cEEG to confirm its findings, as aEEG cannot be used as a substitute for cEEG due to its sensitivity range [10,11].

**Figure 5:**
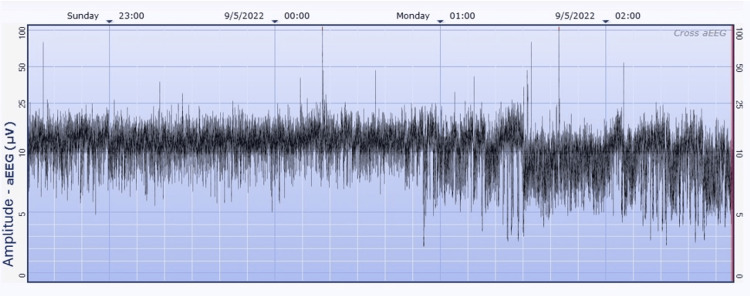
aEEG from a different patient with normal tracings aEEG - Amplitude-integrated electroencephalography

The aEEG emphasizes amplitude differences, whereas the cEEG provides a high-resolution picture of cerebral electrical activity. For a two-channel aEEG, four electrodes are needed, and for a single-channel aEEG, two electrodes are required, with a choice between needle electrodes, gold cups, or hydrogel electrodes. The aEEG electrodes are placed on the left and right parietal regions overlying the apex of vascular watershed areas of the brain, which is an adequate location for detecting abnormalities caused by brain hypoperfusion. The tracing is shown on a highly compressed time scale at a rate of 6 cm/hour, and its interpretation depends on the difference in background activity (bandwidth) [12]. Normal margins for term infants are > 5 mV for the lower border and > 10 mV for the upper edge (Figure 5). A continuous aEEG with amplitudes between 10 and 25 µV, featuring smooth or cyclic variations, generally indicates normal brain function. In this pattern, background activity ranges from 7-10 µV minimum to 10-25 µV maximum. Discontinuous patterns, characterized by alternating low-voltage and higher-amplitude bursts, are common in preterm infants and may suggest brain immaturity or mild encephalopathy. Burst suppression patterns, which have a minimum amplitude of 0-1 µV and bursts exceeding 25 µV, indicate severe brain dysfunction and are linked to poor neurological outcomes. Low voltage patterns exhibit very low voltage, around or below 5 µV, while inactive or flat patterns show primarily isoelectric tracing with background activity below 5 µV [13].

On aEEG, neonatal seizures are shown as an abrupt, transient, sharp rise in the lower and upper margins, often followed by a short period of bandwidth narrowing. The seizure must be at least 30 seconds long and at least 2 mV in amplitude to be detectable. On cEEG, the seizures may present as a sudden appearance of an abnormal electrical event, usually lasting 10 seconds or more, with evolving, repetitive waveforms that gradually build up and then decline in frequency and morphology [14]. It is important to highlight that beyond its use in HIE, aEEG is also beneficial for assessing seizures and monitoring brain activity patterns in a variety of conditions, including intraventricular hemorrhage (IVH), stroke, and other causes of neonatal encephalopathy, further demonstrating its broad applicability in preventing long-term neurological sequelae.

The American Clinical Neurophysiology Society has noted in its guidelines that aEEG can serve as a valuable initial adjunct to cEEG monitoring in detecting neonatal seizures. However, aEEG has several limitations. The potential for underestimating electrographic seizures and its inability to detect brief or low-amplitude seizures and those occurring outside the brain regions covered by the intra-scalp electrodes. Clinicians should be aware that antiepileptic, sedative, and anesthesia agents can influence aEEG background patterns, presenting a limitation in its interpretation. Another limitation is the potential for aEEG pattern misinterpretation due to artifact signals from movement, such as during high-frequency oscillator ventilation, electrocardiography, or extracorporeal membrane oxygenation [5,10,15,16]. 

## Conclusions

In this article, we reviewed the utility of aEEG in detecting subclinical seizures in neonates with HIE during TH. The aEEG is easily accessible and allows prompt treatment in NICUs where cEEG is out of reach upon admission. The aEEG is an excellent screening tool that identifies neonates who need a cEEG.
